# Hempseed Hydrolysates Exhibit Antioxidant Activity in Meat Systems

**DOI:** 10.3390/foods14101728

**Published:** 2025-05-13

**Authors:** Brynn Angeletti, Duy Thinh Trinh, Vermont Dia, Sara Burns, Mary Anna Chester, Rebecca E. Bergee, Tong Wang

**Affiliations:** 1 Department of Food Science, University of Tennessee, 2510 River Dr, Knoxville, TN 37996, USA; 2 Office of Innovative Technologies, Research Computing Support, University of Tennessee, Knoxville, TN 37996, USA

**Keywords:** hempseed meal (HM), hempseed protein isolate (HPI), oxidation, meat systems, antioxidant efficacy, peroxide value (PV), thiobarbituric acid reactive substances (TBARS)

## Abstract

Hempseed hydrolysates prepared by enzymatic hydrolysis have been previously shown to have potent antioxidant activity. The objective of this study is to examine lipid oxidation in beef and turkey meatballs in the presence of selected hempseed hydrolysate products. Alcalase hydrolyzed hempseed meal (AHM10) and hempseed protein isolate (AHPI10) were incorporated into meat products to determine their effects on oxidation over time. Changes in lipid oxidation levels over a 14-day period at 4 °C were determined using peroxide value (PV) and thiobarbituric acid reactive substance (TBARS) methods. Additionally, sensory analyses of the cooked beef and turkey meatballs were performed on day 1 and day 14 of storage to determine the effect of hempseed hydrolysates on the sensory attributes of both meat systems. Overall, AHM10 at 0.4% acted most effectively in beef meatballs and showed enhanced antioxidant activity when compared to EDTA at 100 ppm. Specifically, its use maintained PV below 5.0 meq hydroperoxides/kg oil and TBARS below 1.8 mg MDA/kg oil across the 14-day period. In sensory analysis, no significant differences were found amongst the treatments for various attributes and panelists did not detect bitterness or off flavors. Thus, AHM10 is applicable in food systems as an alternative antioxidant to replace synthetic ones.

## 1. Introduction

Meat products are of high concern for lipid oxidation and off-flavor generation due to their high lipid content, specifically unsaturated fatty acids in phospholipids and triacylglycerols [1,2,3]. Previous studies have suggested that oxidation in lipids is not only proportional to the degree of unsaturation in fatty acids but also related to the physical arrangement of the lipids present [4,5]. Lipid oxidation rates escalate due to the presence of various catalysts such as light, oxygen exposure, and metal ions [3] to generate primary lipid hydroperoxides. The decomposition of hydroperoxides into secondary oxidation products generates rancid off flavors and odors [5,6,7]. Oxidation and quality deterioration of meat products decrease consumer acceptability as sensory attributes become altered [3,8,9], additionally causing changes in nutritional value [4,7,10] and reduction in shelf life [1,3,9].

Red meat such as ground beef contains high concentrations of myoglobin which can be oxidized to produce reactive oxygen species (ROS) during ferrous oxymyoglobin oxidation and is positively related to the generation of thiobarbituric acid reactive substances (TBARSs) [2]. Heme iron is disrupted at high temperatures causing the release of free iron from the porphyrin ring which is known as heme iron shedding [11]. It has been previously determined that free iron ions are the prominent prooxidant substance and can oxidize lipids through Fenton-like reactions [11] with the formation of lipid hydroperoxides which degrade into secondary oxidation products such as aldehydes and ketones causing undesirable odor and flavor [7,12,13]. Additionally, iron is released from the heme as heme dissociates from myoglobin due to the interference of non-covalent interactions during high-temperature conditions which is thought to enhance lipid oxidation [2,5]. This process is highly dependent on the affinity of myoglobin to the heme as lower affinity such as that of metmyoglobin is related to greater ability to promote lipid oxidation [2,5]. Higher concentrations of iron in both free and bound forms [14] are found in red fibers of meat due to a higher amount of myoglobin which increases their susceptibility of lipid oxidation [2]. Beef has been found to have the strongest peroxide-forming potential due to its high iron and myoglobin content [15].

Poultry meat is high in unsaturated fatty acids which oxidizes more readily than saturated fats [1,13] as hydrogen atoms are more easily removed from polyunsaturated fatty acids [6,13]. Oxidation in turkey meat has been found to be highest when compared to other types of poultry, potentially due to its low naturally occurring α-tocopherol content, a fat-soluble antioxidant, within turkey muscle [1,6] as well as its low pH [6].

Ground meats have high susceptibility to lipid oxidation due to the disruption of the cell membrane [7], incorporation of oxygen during grinding, and an increase in surface area due to reduction in particle size [2]. The direct addition of antioxidants such as α-tocopherol in meat is one common strategy in which lipid oxidation can be controlled [5] and has been performed in previous studies examining antioxidant capacity of natural sources in meat systems [16]. Antioxidants act by interfering with the production of free radicals to generate more stable products and decrease the rate of oxidation [3] or by chelating prooxidant ions. Due to the high demand for the use of natural additives in food products by consumers and the potential carcinogenic and toxicological effects of synthetic additives [3], the replacement of synthetic antioxidants such as butylated hydroxyanisole (BHA) and tert-butyl hydroxyquinone (TBHQ) with natural alternatives has become a growing trend in the food industry [5,12]. Naturally sourced antioxidants are of greater desirability as an alternative to synthetic antioxidants due to their comparable efficacy and the safety concerns of synthetic antioxidants [10].

It has been found that natural antioxidants of golden thread extract, clove extract [17], and young kiwifruit polyphenols [15] are beneficial when incorporated into meat systems as they have good solubility and comparable antioxidant activity to synthetic antioxidants [18]. The incorporation of such antioxidants in meat systems has been proven effective in limiting lipid oxidation [19] through mechanisms such as free radical scavenging, chelating metal ions [13], and donating electrons [12]. Plant extracts contain various compounds such as polyphenolics which are bioactive and have been found to exhibit strong antioxidant properties [3,13]. One limitation to using natural antioxidants from plant sources is that they may induce negative sensory attributes in flavor or odor [5], which makes it pertinent that sensory analysis is performed during oxidation studies.

An important group of naturally sourced antioxidant alternatives includes protein hydrolysates obtained from plant and animal proteins [20,21]. Enzymatic hydrolysis enables plant proteins to be broken into smaller peptides that display bioactivity such as inhibiting lipid oxidation. Various protein hydrolysates that have exhibited antioxidant activity in meat systems include rice protein hydrolysates [22], hydrolyzed potato protein [23], milk protein peptides [19], and whey and soy protein hydrolysates [24]. There are numerous mechanisms in which these antioxidant peptides act to inhibit oxidation such as having reducing power and free radical scavenging ability [23]. Hempseed (*Cannabis sativa* L.) protein hydrolysates, specifically hempseed meal (HM) and hempseed protein isolate (HPI) extracted at a pH 10 and hydrolyzed by Alcalase to generate AHM10 and AHPI10 hydrolysate products, were previously studied in an antioxidant screening and in nanoemulsion systems under accelerated oxidation conditions [25]. This study has shown that AHM10 and AHPI10 were effective in inhibiting oxidation in both an antioxidant screening and in nanoemulsion systems. These two hempseed hydrolysates were selected to be further incorporated into meat model systems to determine their effect on oxidation over time and on flavor perception.

The hypothesis of this study is that hempseed hydrolysates act as antioxidants when incorporated into meat systems without imposing any negative sensory attributes to the meat systems. Fully cooked beef and turkey meatballs incorporated with AHM10 and AHPI0 hempseed hydrolysates are analyzed over a 14-day period to examine the changes in lipid oxidation by peroxide value (PV) and thiobarbituric acid reactive substances (TBARS) to measure primary and secondary oxidation products, respectively. AHM10 and AHPI10 at a 0.4% concentration are directly mixed into the beef and turkey meatballs during preparation and oxidation levels are compared to synthetic antioxidants such as EDTA.

## 2. Materials and Methods

### 2.1. Materials

Hempseed hearts were purchased from Food to Live, Inc. (Brooklyn, NY, USA). Hydrolyzed hempseed meal (HM), a residual after protein extraction at pH 10, and hempseed protein isolate (HPI) extracted at pH 10 were prepared according to a previous study [26] by Alcalase (A) hydrolysis. Hydrolysis of HM and HPI generated AHM10 and AHPI10. Protein content of HM10 was 71.74 ± 0.45% and that of HPI10 was 74.79 ± 0.14% [25]. Average molecular weight of AHM10 was 3732 ± 691 Da and that of AHPI10 was 3781 ± 1378 Da [25]. Ground beef (80% lean, 20% fat) and ground turkey (85% lean, 15% fat) were purchased from Publix grocery store (Knoxville, TN, USA). Alcalase was purchased from EMD Millipore Corp. (Billerica, MA, USA). The Gas Chromatography (GC) standard FAME mixture used was Nu-Chek-Prep standard 15A containing methyl palmitate (16:0), methyl stearate (18:0), methyl oleate (18:1), methyl lineolate (18:2), methyl linolenate (18:3), and methyl arachidate (20:0) (Elysian, MN, USA). All reagents and chemicals were purchased from Thermo Fisher Scientific (Ward Hill, MA, USA) and Sigma-Aldrich (St. Louis, MO, USA).

### 2.2. Preparation of Turkey and Beef Meatballs Using Hempseed Hydrolysates

#### 2.2.1. Hydrolysis of Hempseed Meal and Protein Isolate by Alcalase to Generate AHM-10 and AHPI-10 Samples

The Alcalase enzyme was used to hydrolyze HM10 and HPI10 based on previous antioxidant screening and nanoemulsion results [25]. Preparation of hydrolyzed HM and HPI was performed according to a previous study [26]. To hydrolyze the prepared HM10 and HPI10 samples, a 50 mL centrifuge tube was used to suspend 2.5 g of HM10 and HPI10 in 25 mL of DI water at Alcalase’ optimum pH (8.0), and it was stirred in a cold room overnight. HM10 and HPI10 suspensions were then placed in a water bath at Alcalase’ optimum temperature (55 °C) to equilibrate before adding the enzyme to the suspension at a concentration of 0.1% *w*/*v*. The HM10 and HPI10 suspensions were hydrolyzed by Alcalase for 30 min, boiled for 10 min to inactivate the Alcalase enzyme, and then placed in the freezer at −40 °C. After freezing, AHM10 and AHPI10 samples were lyophilized for 7 days. Both AHM10 and AHPI10 were used as is in the meat systems.

#### 2.2.2. Beef and Turkey Meatball Formulation and Preparation

Approximately 13,000 g of ground beef or ground turkey were each mixed with 1.5% salt (*w*/*w*). The negative control meatball only contained beef or turkey and salt while the positive control additionally contained 100 ppm ethylenediaminetetraacetic acid (EDTA) disodium salt dihydrate as a metal-chelating agent. EDTA was utilized to show the metal-chelating efficacy of hempseed hydrolysate which has been previously reported and compared to EDTA [25]. The meat was separated into 3250 g sections and each antioxidant treatment was added. Meatballs were made with the addition of 0.4% (as is) AHM10 or AHPI10. The concentration of 0.4% hydrolysate incorporation into ground meats was according to the usage level identified in preliminary trials. One batch of each meat type was prepared. Each meatball was weighed out in 28 g portions and rolled into a ball shape. The raw beef and turkey meatballs were cooked at 400 °F (204 °C) for 23–30 min in a convection oven until reaching an internal temperature of 160–165 °F (71–74 °C). Meatball samples were then cooled, cut into quadrants, and placed in oxygen-permeable polyethylene bags that were then stored in the refrigerator at 4 °C for 14 days. Samples were taken for lipid extraction for oxidation measurements every other day and the extracts were stored in the freezer for oxidation analysis. Sensory analysis was completed on day 1 and day 14 storage of the cooked meatball. For each treatment, 3 meatballs were made as 3 replicates.

### 2.3. Quantification of Lipid in Beef and Turkey Meatballs

#### 2.3.1. Moisture Content

About 1 g of cooked control meatball was placed in an aluminum dish and the moisture content of beef and turkey was analyzed using infrared moisture analyzer OHAUS Mb45 (Parsippany, NJ, USA) at 100 °C for 15 min. The beef and turkey were analyzed in triplicate on day 0 and day 14 of oxidation testing.

#### 2.3.2. Quantification of Total Lipids

Lipid quantification was completed according to the Folch extraction method. About 25 g of raw beef and turkey meatball were broken up and placed in separate 200 mL glass beakers. Exactly 100 mL of 2:1 chloroform/methanol was added to each beaker and stirred for 1 h using a stir bar. After 1 h, the meat suspension was filtered using vacuum filtration with 2 mm filter paper relative to the total volume. The exact volumes of each filtrate were determined and 20% of water was added to the filtrate in a separatory funnel. The filtrate and water were mixed and allowed to separate overnight. The lower organic phase of the filtrates was rotary evaporated (Buchi; New Castle, DE, USA) using a 60 °F water bath, 100 rpm spin, and at 300 mbar pressure. The lipid weight of the beef and turkey were determined gravimetrically. The percentages of lipid from ground beef and turkey were then calculated by dividing the lipid weight determined by the initial weight of the ground beef or turkey meat and multiplying the value by 100. Lipid quantification was performed in triplicate for each meat type.

#### 2.3.3. Fatty Acid Composition Determination of Beef and Turkey Lipids Using GC

Prior to analysis of fatty acid composition by GC, lipids were interesterified to form fatty acid methyl esters (FAMEs). Exactly one drop of beef or turkey lipid produced from quantification was placed into a capped glass test tube. About 1–2 mL of sodium methoxide (1 M) was added to each lipid sample. The test tubes were sealed with Teflon tape, capped, and then placed in the oven at 60 °C for 2 h to allow for the interesterification reaction to occur. Following interesterification, a few drops of water and 2 mL of hexane were added to each test tube to allow for phase separation to occur. If more phase separation was needed, sodium chloride (NaCl) was added to accelerate the process. The fatty acid methyl esters separated into the hexane layer and approximately 1.5 mL was extracted from the top layer. The FAME extracts for beef and turkey were subjected to GC analysis.

Fatty acid composition was determined using capillary GC (Shimadzu, Kyoto, Japan) with a flame ionization detector and HP-88 column (100 m × 0.25 mm × 0.20 μm). Separation and detection of fatty acid methyl esters were performed based on preliminary trials with the following temperature program: initial temperature of 100 °C held for 2 min, rate increase of 10 °C/min to a final temperature of 230 °C, held 5 min, with total analysis time of 20 min. The injector and detector temperatures were 180 °C and 250 °C, respectively, and the split ratio was 10. A volume of 1 μL was injected and the carrier gas was helium at a total flow of 15.6 mL/min and purge flow of 3.0 mL/min. The fatty acid identity was determined by comparing the chromatogram peaks of the beef and turkey lipids to that of a standard FAME mixture and canola oil FAME as a standard. Fatty acid composition percentages were determined by dividing the peak area by the total area of the peaks and multiplying by 100. GC was performed in triplicate for both beef and turkey lipids.

### 2.4. Oxidation Quantification of Beef and Turkey Meatballs

#### 2.4.1. Lipid Extraction from Beef and Turkey Meatballs

Lipid extraction of the turkey and beef meatball samples was performed immediately after sampling according to the Folch method with some modifications. About 1.5–2 g of each meatball sample was taken and accurately weighed and placed in a 15 mL glass test tube. Following sampling of the meatball, 10 mL of chloroform/methanol solvent was added to each meat sample and allowed to soak for 4 h. Samples were then centrifuged for 5 min at 1000 rpm and 3 mL of lipid extract was then taken from the solvent layer of the meat dispersion. Lipid extract samples were placed in the freezer at −18 °C for further oxidation product quantification. Meatball treatments in triplicate were analyzed twice for oxidation analyses.

#### 2.4.2. PV Determination by the Ferrous Thiocyanate Method

PV by the ferric thiocyanate method quantifies the concentration of primary oxidation products, specifically lipid hydroperoxides, through a colorimetric method. This method quantifies the oxidation of ferrous iron to ferric iron by hydroperoxides and allows for a red color to generate. Potassium thiocyanate solution (3.94 M) was prepared in HPLC water, and a ferrous sulfate solution (35.9 mM) was made by dissolving ferrous sulfate pentahydrate in HPLC water followed by the addition of 1 mL of 10 N hydrochloric acid (HCl). All reagents were deoxygenated by purging with nitrogen gas. The cumene hydroperoxide (CuOOH) standard curve was prepared using a 100 μM stock solution and standard solutions were made by adding varying volumes of CuOOH stock solution to different volumes of 2:1 chloroform/methanol achieving a concentration range of 0–10 μM. Additionally, an aliquot (100 μL) of each lipid extract was taken and diluted by 2 mL of CHCl_3_/MeOH to achieve a final lipid concentration below 5 mg/mL. A reagent blank was prepared using 2 mL CHCl_3_/MeOH. Following standard curve and sample preparation, potassium thiocyanate (12 μL) and ferrous sulfate solution (12 μL) were added, respectively, to each sample or standard with vortexing in between their addition. Samples were then incubated in the dark for 20 min at room temperature and the absorbance was read at 510 nm using a UV–spectrophotometer against the reagent blank. Using the CuOOH standard curve, peroxide values were calculated and reported in milliequivalents (meq) of hydroperoxides per kilogram (kg) of oil. Three replicates of each beef and turkey lipid extract treatments were analyzed and each assayed twice.

#### 2.4.3. TBARS Determination

The TBARS assay measures the secondary oxidation products, mainly but not specific to malondialdehyde (MDA), which is a compound responsible for off flavors and odors in food products [27]. A pink colored complex is generated through a heated reaction under acidic conditions between thiobarbituric acid (TBA) and MDA present in a sample. Reagent preparation included butylated hydroxy anisole (BHA) (7.2% in ethanol) and a TBA/trichloroacetic acid (TCA) solution (20 mM TBA/15% TCA). Standard solutions were prepared using malondialdehyde bis (dimethyl acetal) in a range of 0–0.4 μM using varying volumes of MDA and DI water. Samples were prepared by adding 250 μL of meat lipid extract to 225 μL of DI water. A blank was also prepared using 250 μL DI water. BHA (12.5 µL) and TBA/TCA solution (500 μL) were added to standard and sample solutions with vortexing in between their addition. The mixtures were vortexed again and incubated in a water bath for 15 min at 90 °C allowing for the color reaction to occur. Samples were cooled for 10 min in cold water and then centrifuged at 3000× *g* for 15 min at 4 °C. The absorbance of samples was then read at 531 nm against the blank solution. Using the MDA standard curve, TBARS values were calculated and reported in milligram (mg) MDA per kg of oil. Three replicates of each beef and turkey lipid extract treatment were analyzed, each assayed twice.

#### 2.4.4. Statistical Analysis

Descriptive statistics were calculated by treatment and time for PV turkey, PV beef, TBARS turkey, and TBARS beef. Repeated measures mixed-model analysis of variance (ANOVA) was used to evaluate each outcome for time, treatment, and treatment-by-time difference. A linear trend effect was tested across time using a custom contrast post hoc comparison and tested per treatment for models with a significant interaction. Normality of residuals and equality of variance were evaluated for all models using the Shapiro–Wilk test, Q-Q plots, Levene’s test for equality of variances, boxplots, and studentized residual diagnostics. An outlier was detected due to measurement error in the PV turkey control group on day 2 and was removed for statistical analysis. All models required a rank transformation to meet the normality assumption. Heterogeneous between-group variance structures were incorporated into all models to adjust for unequal group variances. Bonferroni *p*-value adjustments were applied to linear trend post hoc tests across time per treatment. Dunnett’s *p*-value adjustments were applied to post hoc tests comparing treatments to the control group. After transformations and heterogenous variances were applied, all statistical assumptions were met for all models. A *p*-value less than 0.05 was considered significant. Statistical analysis was performed using commercial software (SAS software, Version 9.4, Cary, North Carolina, Release TS1M8).

### 2.5. Sensory Analysis of Beef and Turkey Meatballs

#### 2.5.1. Paired Comparison Discrimination Sensory Evaluation for “Fresh” Meatballs

A 2-Alternate Forced Choice (2-AFC) paired comparison sensory evaluation by about 80 blind, untrained panelists was performed on day 1 of refrigerated storage of cooked beef and turkey meatballs. Meatball samples were reheated at 135 °C for 5 min and then stored in warming bins prior to serving. Meatball samples were placed in plastic cups and randomly labeled with a three-digit number. The 2-AFC test on day 1 included the control sample, EDTA treatment, and both hempseed hydrolysate containing meatball samples. In the test, each participant compared the meatiness of all possible pairs of the beef and turkey meatballs. Following comparison of meatiness, a Choose-All-That-Apply (CATA) was prompted for panelists to choose which attributes were different among treatments. Samples were provided with a cup to dispose in, water to wash with between samples, and crackers to cleanse the palette. Beef and turkey samples were analyzed on separate days, and each was completed in the same day.

#### 2.5.2. Consumer Acceptance Sensory Evaluation

On day 14 of meatball storage, 100 blind, untrained panelists completed sensory evaluation of a consumer acceptance test using a 9-point hedonic scale (1—dislike extremely and 9—like extremely) as well as a line scale for intensity (0—not intense at all and 15—extremely intense) on various parameters describing the beef and turkey meatball treatments. Various attributes being rated for acceptability included aroma, appearance, texture, or mouthfeel and overall liking. The intensity line scale ranked flavor intensity and off flavor. Additional questions following the hedonic scale test were related to whether the panelist would eat the meatball products again. Meatball samples were reheated at 135 °C for 5 min and then stored in warming bins prior to serving. The samples were provided in plastic cups and randomly labeled with a three-digit number. Samples were provided with a cup to dispose in, water to wash with between samples, and crackers to cleanse the palette. Beef and turkey samples were analyzed on separate days, and each was completed in the same day.

#### 2.5.3. Statistical Analysis of Sensory Tests

The determination of multiple sensory attributes on the 9-point hedonic scale for appearance, aroma, meat flavor, texture, and overall opinion was conducted. Analysis of the attributes and the intensity line scale were evaluated for treatment differences using ANOVA and the means and standard deviation of responses are reported. Tukey–Kramer *p*-value adjustments were applied to post hoc tests to identify the specific differences between treatment groups. A *p*-value less than 0.05 was considered significant. Statistical analysis of sensory tests was performed using RedJade Sensory Software (RedJade Sensory Solutions LLC, Martinez, CA, USA).

## 3. Results and Discussion

### 3.1. Moisture Content, Total Lipid Quantification, and Fatty Acid Composition of Ground Beef and Turkey

The content of total lipid and moisture of both beef and turkey meatballs after cooking and storage can be seen in Table 1. The loss of moisture was accounted for in calculations of PV and TBARS values. For beef meatballs, the total lipid was about 19.0 ± 0.5% (as-is basis), which was expected as the ground beef being used was labeled as 80% solid and 20% fat. It was also determined that the total lipid of turkey meatballs was 14.9 ± 3.3% (as is basis) which again was expected as the ground turkey purchased was 85% solid and 15% fat. For beef meatballs, the moisture content decreased from 18.3 ± 4.3% to 12.7 ± 0.6% while the turkey meatballs decreased in half in moisture content from day 0 (33.6 ± 6.0%) to day 14 (15.1 ± 3.3%). The difference in matrix properties may be the cause for such a different rate of moisture loss.

Additionally, the fatty acid composition of both ground beef and turkey was determined to understand the fatty acid profile of both meat systems and potentially explain their susceptibility to oxidation. In Table 2, it can generally be observed that beef had higher percentages of saturated and monounsaturated fatty acids but lower percentages of polyunsaturated fatty acids compared to turkey. Beef’s total saturated fat content is 44.4% compared to turkey meat at 27.2%, which was expected as turkey meat is known to have higher amounts of polyunsaturated fatty acids. It was unexpected that for both beef and turkey, α-linolenic acid (18:3) was not able to be quantified when compared to the standard mixture.

When comparing the fatty acid composition of beef to previous literature it was found that the levels of 14:0 (mystric acid), 16:0 (palmitic acid), and 16:1 (palmitoleic acid) were similar to what was previously found [28]. Additionally, it was determined that there was 26.4 ± 0.2% palmitic acid in the ground beef while perirenal meat had 24.7% and subcutaneous meat had 24.1% [28]. Previous literature also reported 25% palmitic acid in grain-fed crossbred steers [29], which is similar to this study. The palmitoleic acid (4.0 ± 0.2%) and linoleic acid (2.9 ± 0.1%) quantities were closer to the subcutaneous cut of meat at 4.3% and 2.1%, respectively, in comparison to perirenal cuts [28]. Additionally, grass-fed Simmental bulls were determined to have 2.2% linoleic acid present in their meat [29], similar to what was determined in this study. The major difference found in previous literature is that our 18:1 (oleic acid) percentage (42.9 ± 0.3%) is much higher than what has been previously found in both perirenal (23.5%) and subcutaneous (35.3%) cuts of meat [28]. This difference in oleic acid may be due to environmental and feed factors. For trans fatty acids present in beef, it was determined that the ground beef had about 5.8 ± 0.1% of 18:1t (vaccenic acid) while previous literature reported 3.2% and 2.3% for grass- and grain-fed Angus steers, respectively [29].

The fatty acid composition of turkey can also be seen in Table 2. One major difference when compared to ground beef is that turkey meat had a much higher value of linoleic acid, which was expected. When comparing to previous literature, the mystric acid composition (1.0 ± 0.2%) was similar to what was found in raw (1.1%) and uncured (1.2%) turkey breast meat [30]. The palmitic acid found (19.7 ± 0.2%) was higher than one publication that had 12.6% [31] but was also lower than a previous publication in raw (24.9%) and uncured (29.1%) turkey breast meat [30]. The palmitoleic acid determined (3.2 ± 0.3%) fell between the two previous publications [30]. The stearic acid found (6.4 ± 0.6%) was about 2% higher than what was previously found (4.1%) [31] but was also 2.5% point lower than reported in another study [30]. Oleic acid (34.28 ± 1.09%) was higher than what was previously found in raw (29.3%) turkey breast meat but was comparable to that found in uncured (33%) turkey breast meat [30]. Additionally for linoleic acid, there was a slight difference compared to what was previously found [31]. However, the arachidic acid (20:0) found (2.8 ± 0.3%) was much higher than what was found in raw (0.3%) and uncured (0.1%) turkey breast meat [30] as well as in another publication on turkey meat (0.3%) [31]. These variations in the fatty acid composition of ground turkey meat in comparison to other publications may be due to feed and environmental factors [30,31].

### 3.2. PV of Beef and Turkey Meatballs Under 14-Day Refrigerated Conditions

**Figure 1:**
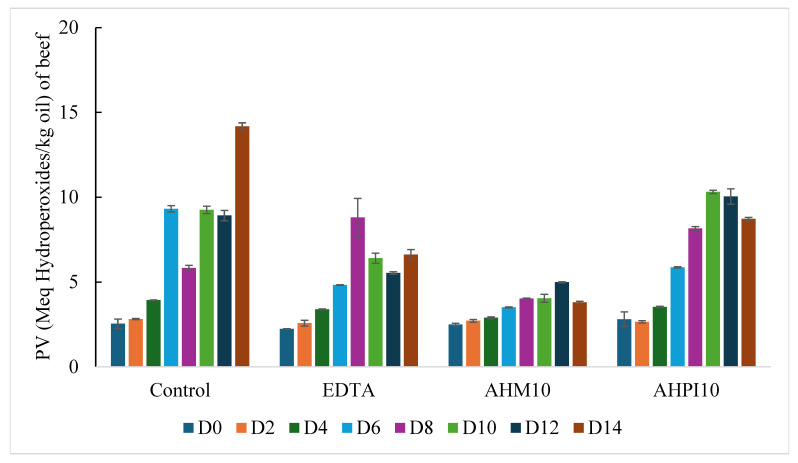

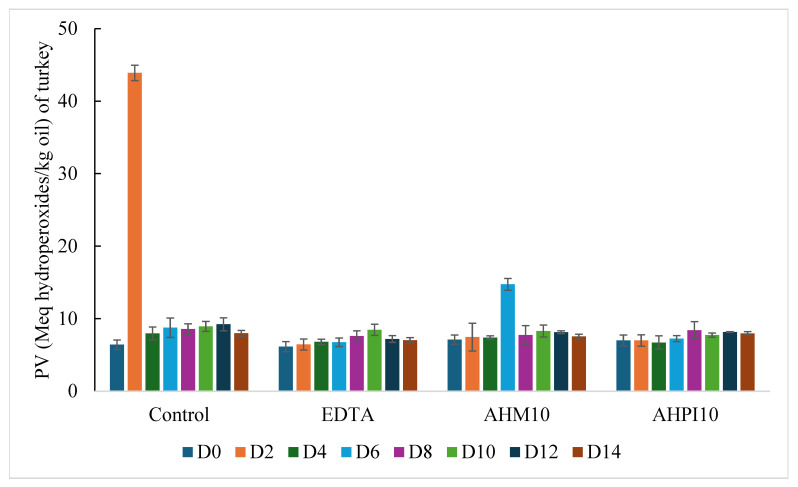
PV of four beef (**top**) and turkey (**bottom**) meatball treatments oxidized for 14 days at 4 °C. D = day.

The increase in primary oxidation products in beef meatballs over a 14-day period can be observed in Figure 1 The *p*-values for treatment, time, and interaction of treatment and time for PV assay are shown in Table 3. A significant linear trend was found across the 14-day period (*p*-value < 0.001) for beef meatballs. When comparing the treatments against the control, the EDTA (*p*-value < 0.05) and the AHM10 (*p*-value < 0.01) treatments were significantly lower. Overall, it can be clearly observed that the control meatball had the highest peroxide value of 14.2 ± 0.2 meq hydroperoxides/kg oil by day 14 when compared to the other treatments. There was a general increase in PV over the 14 days for all beef meatball treatments, but the addition of EDTA at 100 ppm, a synthetic antioxidant, and the two selected hempseed hydrolysates used at 0.4% were effective in preventing oxidation when compared to the control. EDTA reached a high value of 8.8 ± 1.1 meq hydroperoxides/kg oil on day 8 and then decreased by day 14. When comparing the two hempseed hydrolysates to the EDTA containing beef meatball, it was clear that AHM10 performed much better than both AHPI10 and the EDTA treatments. AHM10 reached a high value of 5.0 ± 0.0 meq hydroperoxides/kg oil on day 12 and then decreased to 3.8 ± 0.1 meq hydroperoxides/kg oil on day 14. On the contrary, AHPI10 did not perform better than EDTA reaching a high value of 10.3 ± 0.1 meq hydroperoxides/kg oil on day 10 and also saw a decrease by day 14. By day 14 of storing beef meatballs, all treatments had PV values over 3.8 ± 0.1 meq hydroperoxides/kg oil, with the lowest being the AHM10 containing meatball as seen in Figure 1. For all treatments besides the control, the general trend of increasing PV then decreasing by day 14 was expected as primary oxidation products begin to decompose into secondary products. Overall, it can be concluded that AHM10 when used at 0.4% worked more effectively than EDTA at 100 ppm, a known metal chelator, to inhibit oxidation in beef meatballs across a 14-day period.

In previous literature, cooked beef patties containing 4% Alcalase hydrolyzed potato protein exhibited PV values of 2.8 meq hydroperoxides/kg sample on day 7 of refrigerated storage [23]. This value is comparable to those found in AHM10 on day 4 of oxidation at 2.9 ± 0.1 meq hydroperoxides/kg oil. Another study using casein protein hydrolysates from 2% to 8% exhibited PV values between 0. 8 ± 0.0 and 0.7 ± 0.0 meq hydroperoxides/kg oil, respectively [20], considerably lower than those exhibited by all treatments in beef meatballs. This may be due to the higher concentrations (2–8%) of casein protein hydrolysates used in comparison to the concentration in which hempseed hydrolysates were used (0.4%) in this study.

The peroxide value of turkey meatballs can be observed in Figure 1. The *p*-values for treatment, time, and interaction of treatment and time in the PV assay can be seen in Table 3. Again, a significant linear trend was found across the oxidation day (*p*-value < 0.001), although this may not be practically significant. When comparing the treatments against the control, the EDTA (*p*-value < 0.001) and the AHPI10 (*p*-value < 0.01) treatments were significantly lower. Two potential outliers were present seen in the control meatball on day 2 (43.9 ± 1.1 meq hydroperoxides/kg oil) and AHM10 on day 6 (14.7 ± 0.8 meq hydroperoxides/kg oil) which was removed during statistical analysis. When comparing turkey to beef, beef had lower initial PV values but increased more than turkey by the end of the 14-day period. This may be due to the fact that there is a pro-oxidant effect of heme iron in red meat that contributed to a greater increase in PV values in beef [14]. Ground turkey meat was also expected to oxidize more than beef due to the high linoleic polyunsaturated fatty acid content, but this did not take place potentially due to unknown naturally occurring antioxidants present in the lipid that made turkey more oxidatively stable than the beef. The EDTA, AHM10, and AHPI10 treatments seemed to maintain relatively similar peroxide values with EDTA reaching a high of 8.5 ± 0.8 meq hydroperoxides/kg oil on day 10. Again, the PV products seemed to slightly decrease by day 14 in all treatments after reaching its highest value. Though it was expected that AHM10 would perform more effectively than both EDTA and AHPI10, it seemed that AHM10 acted comparably to the two treatments, which still shows that AHM10 is a valuable natural antioxidant alternative when compared to synthetic antioxidants in food systems.

### 3.3. TBARS of Beef and Turkey Meatballs Under 14-Day Refrigerated Conditions

**Figure 2:**
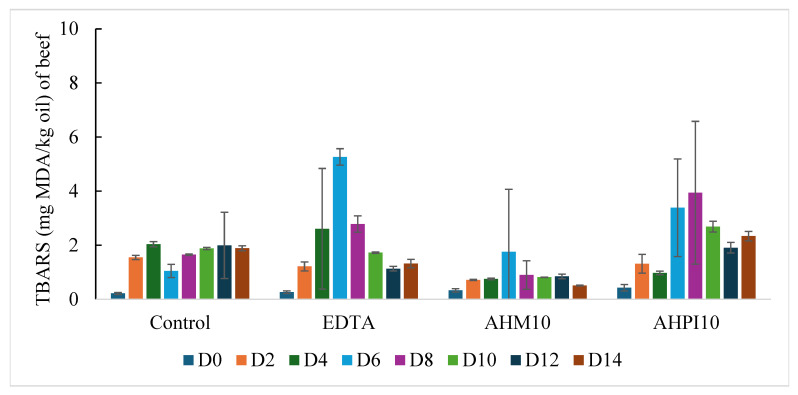

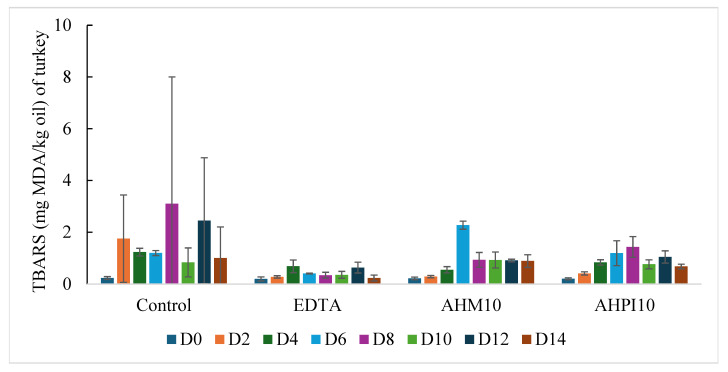
TBARS of four beef (**top**) and turkey (**bottom**) meatball treatments oxidized for 14 days at 4 °C. D = day.

The secondary oxidation products in beef meatballs over a 14-day period can be observed in Figure 2. The *p*-values for TBARS are shown in Table 3. Large standard deviations were found in the TBARS data potentially due to sampling of a heterogenous meat mixture as well as the known interferences that affect TBARS quantification such as amino acids [32]. Treatments EDTA (*p*-value < 0.05), AHPI10 (*p*-value < 0.001), and the control (*p*-value < 0.001) were found to have a significant linear trend at the early stage of the 14-day period. When compared to the control, only the AHM10 treatment had significantly lower TBARS than the control (*p*-value < 0.01). Unexpectedly, AHPI10 and EDTA treatments exhibited TBARS values higher than that of the control. EDTA reached a high of 5.3 ± 0.3 mg MDA/kg oil on day 6 while AHPI10 reached 3.9 ± 2.6 mg MDA/kg oil on day 8. Thus, AHM10 seemed to work most effectively in inhibiting the decomposition of primary products into secondary products as the TBARS values maintained levels below 1.8 ± 2.3 mg MDA/kg oil. Low TBARS values indicate a low level of rancidity, which can be further exemplified through sensory analysis data when examining the absence of off flavors on day 14 in AHM10 beef meatballs. Overall, AHM10 has shown its ability to act as a natural antioxidant in beef systems when compared to a synthetic antioxidant by preventing oxidation from progressing.

Previous literature examining the TBARS values of ground beef with the incorporation of protein hydrolysates have reported similar values of secondary oxidation products. For example, cooked beef patties containing 4% potato protein hydrolysates prepared by Alcalase inhibited secondary oxidation products with 1.3 mg MDA/kg sample across a 7-day period when stored at 4 °C [23]. These values are comparable to that seen in beef meatballs across all treatments but especially in AHM10 meatballs. Beef nuggets containing 8% casein protein hydrolysates exhibited a TBARS value of 0.4 ± 0.0 mg MDA/kg sample on day 15 of storage [20]. This value is lower than what was found on day 14 of hempseed hydrolysates in ground beef, but is likely due to the high concentration of casein protein hydrolysate used.

The secondary oxidation products of turkey meatballs can be seen in the bottom graph of Figure 2, and *p*-values are shown in Table 3. A significant linear trend was found across the days, particularly at early stage of the storage (*p*-value < 0.001), albeit large standard deviations. As for treatment comparisons, only the EDTA treatment was found to have significantly lower TBARS than the control (*p*-value < 0.001). As seen in the PV assay for primary oxidation products, the secondary oxidation products determined for turkey meatballs are lower those that for beef meatballs. This again was unexpected due to the high susceptibility of polyunsaturated fatty acids to be oxidized, but it may be attributed to the higher free iron and heme iron content in beef and/or higher amounts of antioxidants in turkey. Overall, the control meatball presented the highest TBARS values reaching 3.1 ± 4.9 mg MDA/kg oil on day 8. EDTA performed the most effectively out of all turkey treatments across the 14-day period. AHM10 seemed to perform more effectively than AHPI10 in turkey meatballs. The slight decrease in TBARS values was attributed to the loss of oxidation products such as low-molecular-weight volatile compounds as they can be volatilized and lost during longer storage times [33]. Unfortunately, neither AHM10 nor AHPI10 when used at a 0.4% concentration performed more effectively than EDTA at 100 ppm to inhibit the decomposition of primary oxidation products into secondary products in the turkey meatballs. It is possible that AHM10 is more effective in a beef than in turkey as AHM10 has previously exhibited an ability to act as a strong iron chelator [25], which may have contributed to its ability to perform effectively in an iron-containing food system such as beef.

The TBARS values of turkey meat sausages with the addition of 0.01–0.2% (*w*/*w*) Goby (*Zosterissessor ophiocephalus*) fish protein hydrolysates prepared by various digestive proteases was determined across a 25-day period and was compared against a negative control as well as a 0.2% vitamin C positive control [33]. Overall, the 0.2% Goby fish protein hydrolysates exhibited the lowest TBARS values by the end of the 25-day period. Overall, turkey meat sausages with the incorporation of Goby fish protein hydrolysates had TBARS values below 1.3 mg MDA/kg sample across the 25-day period [33]. Our values are comparable to the Goby fish protein hydrolysate.

### 3.4. Sensory Evaluation of Beef and Turkey Meatballs on Day 1 and Day 14 of Refrigerated Storage

For day 1 sensory evaluation, a 2-AFC paired comparison test was implemented for both beef and turkey meatballs to determine whether there was a difference in meatiness found among treatments. The main finding of the sensory analysis for beef meatballs on day 1, as shown in Table 4, was that there was a borderline significant difference in meatiness found in the control meatball with EDTA (*p*-value = 0.057) and in the AHM10 (*p*-value = 0.057) meatballs when compared to the control. Additionally, there was no difference found when comparing the EDTA meatball with the hempseed hydrolysate containing meatballs, which suggests that in addition to performing comparably to EDTA, the hempseed hydrolysates did not differ in meatiness. After completing the 2-AFC for each pair, panelists were asked to select attributes that they perceived as differing using a Check All That Apply (CATA) system. “No difference” was included in the list of possible attributes. Table 5 presents the *p*-values of each attribute that was determined to be significantly different amongst the treatments. The main attributes that were perceived as differing among samples were savory flavor (*p*-value < 0.0001), mouthfeel (*p*-value < 0.0001), firmness (*p*-value < 0.0001), and meaty flavor (*p*-value < 0.0001). Overall, the data revealed that bitterness and off flavor were not detected in the meatballs when compared to the control. Additionally, for beef meatballs on day 1, color and aroma were not perceptually affected by the addition of hempseed hydrolysates indicating their applicability to meat systems as an alternative to synthetic antioxidants.

For turkey meatballs on day 1, there were significant differences found in the meatiness of the control meatball compared to that containing EDTA (*p*-value < 0.05) as well as when compared to the AHM10 (*p*-value < 0.05) and AHPI10 containing meatballs (*p*-value = 0.001) as shown in Table 4. This indicates a difference was detected between the control and treatments for both the synthetic and natural antioxidants. However, the two hydrolysate treatments were not different from the EDTA treatment. The reason for the two hydrolysate treatments being different from each other cannot be well explained. When comparing sensory CATA attributes seen in Table 5 of the turkey meatballs, the savory flavor (*p*-value < 0.0001), mouthfeel (*p*-value < 0.0001), rancidity (*p*-value < 0.0001), firmness (*p*-value < 0.0001), and meat flavor (*p*-value < 0.0001) were all significantly different.

For the day 14 consumer acceptance test, Table 6 depicts the hedonic mean score out of nine for the attributes tested in each treatment for beef meatballs. This indicates the overall acceptability of each attribute for each treatment. For all attributes, no statistical differences were found. This indicates that none of the test samples significantly differed from the control with respect to any of the attributes of interest, meaning they are equally acceptable. Therefore, it can be concluded that the treatments were all comparable to each other and did not have noticeable differences for the consumer. This indicates that AHM10 and AHPI10 can be incorporated into beef without posing negative consequences to flavor, odor, or appearance.

For turkey meatballs on day 14, Table 6 depicts the mean scores and *p*-values of each attribute for all treatments. Again, no significant differences were found in the data set for day 14 consumer acceptability scores, indicating the equality in acceptability for all treatments. Numerically, the AHM10 meatball seemed to have the highest liking score for appearance (5.34), meat flavor (5.84), texture (5.38), and overall opinion (5.63) when compared to the control, EDTA, and AHPI10.

Table 7 depicts the percentage of people that detected an off flavor in the different treatments for beef and turkey as well as the *p*-value associated with the data. Additionally, the off-flavor intensity of each treatment when scored on a 15-point line scale is also presented. For beef meatballs on day 14, significant differences were found considering the off-flavor presence among the treatments (*p*-value < 0.001). AHPI10 had the highest score with 49% of panelists saying there was an off flavor detected. AHM10 (24%) had the lowest percentage when compared to the control (39%) and EDTA (30%) treatments, indicating that there was less of an off flavor detected in the AHM10 treatment when compared to all other treatments. This also suggests the high acceptability of AHM10 as consumers are less likely to accept a food product that contains an off flavor. As for the off-flavor intensity score for beef meatballs seen in Table 7, there was no significant difference found among treatments. Despite the lack of significant differences, the AHM10 meatball seemingly had the lowest off-flavor intensity when compared to all other treatments with EDTA potentially having the highest intensity value. This further suggests AHM10’s applicability in place of synthetic antioxidants such as EDTA. For turkey meatballs as shown in Table 7, 26% of panelists detected an off flavor in the sample for AHM10 meatballs which is comparable to the control (25%) and EDTA (24%) meatballs. These values may be comparable, but since no significant differences were found amongst the treatments, it can be concluded that the treatments were equally accepted by consumers in terms of off-flavor presence. When comparing the off-flavor intensity of the treatments, there were no significant differences. Despite the absence of significant differences, the AHM10 meatball may have had the highest numerical off-flavor intensity while AHPI10 had the lowest intensity.

Table 7 also depicts the percentage of consumers that would eat each product again. There were no significant differences found amongst treatments for both beef and turkey meatballs, which indicates their equal acceptability. AHM10 seemed to have the highest score when rating whether the consumer would eat the meatball again. For example, 62% of consumers said they would eat the AHM10 meatball again while 53% said they would eat the control again and 49% said they would eat the EDTA meatball again. For turkey meatballs, 59% of consumers may have said they would eat the AHM10 turkey meatball while 53% said they would eat the control again, and 45% said they would eat the EDTA meatball again. This indicates that all treatments were acceptable to the consumers, with AHM10 treatment performing slightly better than others.

In summary, AHM10 is a valuable and effective natural alternative to synthetic antioxidants, such as EDTA, in meat systems, particularly in ground beef. Ground beef and ground turkey had differing fatty acid profiles as well as iron content making them both suitable for an oxidation study. By oxidizing cooked beef and turkey meatballs for 14 days with the incorporation of hempseed hydrolysates, AHM10 performed more effectively than EDTA and AHPI10 to prevent lipid oxidation. Overall, the protein hydrolysate antioxidant treatments were more effective in a beef system compared to turkey, which was attributed to their iron chelating ability and the high abundance of free iron and bound iron in ground beef. Through sensory analyses, it was determined that there were no significant differences found among treatments, suggesting that AHM10 is applicable as an antioxidant without posing any negative effects to flavor or odor. One uniqueness of this study is the demonstration of hydrolysates’ effectiveness at relatively low concentration of 0.4% in ground meat compared to the dosage tested in other studies. Ultimately, AHM10 is a very effective antioxidant in ground beef systems, and it can play a significant role in enhancing oxidative stability of foods by using safe and feasible alternatives to replace synthetic antioxidants as the trend towards natural ingredients continues to rise.

## Figures and Tables

**Table 1:** Total Lipid (%, as-is) and Moisture Content (%) of Beef and Turkey Meatballs After Cooking.

Sample	Total Lipid	Moisture Content (Day 0)	Moisture Content (Day 14)
Beef	19.0 ± 0.5	18.3 ± 4.3	12.7 ± 0.6
Turkey	14.9 ± 3.3	33.6 ± 6.0	15.1 ± 3.3

**Table 2:** Fatty Acid Composition (%) of Beef and Turkey Lipids.

Sample	14:0	16:0	16:1	18:0	18:1t	18:1	18:2	20:0
Beef	3.3 ± 0.2	26.4 ± 0.2	4.0 ± 0.2	14.7 ± 0.3	5.8 ± 0.1	42.9 ± 0.3	2.9 ± 0.1	-
Turkey	1.0 ± 0.2	19.7 ± 0.2	3.2 ± 0.3	6.4 ± 0.6	-	34.3 ± 1.1	32.5 ± 0.9	2.8 ± 0.3

**Table 3:** *p*-Values of Treatment, Time, and Interactions for Peroxide Value (PV) and Thiobarbituric Acid Reactive Substance (TBARS) Assays.

Sample and Assay	*p*-Value for Treatment	*p*-Value for Time	*p*-Value for Interaction
PV for Beef	<0.001	<0.001	0.225
PV for Turkey	<0.001	<0.001	0.147
TBARS for Beef	0.002	<0.001	<0.001
TBARS for Turkey	0.001	<0.001	0.118

**Table 4:** Summary of 2-AFC Sensory Analysis for Meatiness of All Possible Pairs of the Beef and Turkey Meatballs on Day 1.

		EDTA vs. Control	AHM10 vs. Control	AHP10 vs. Control	AHM10 vs. EDTA	AHP10 vs. EDTA	AHM10 vs. AHP10
Beef	% Correct	59	59	52	48	38	43
	*p*-Value	0.057	0.057	0.411	0.674	0.988	0.912
Turkey	% Correct	62	61	68	30	39	65
	*p*-Value	0.021	0.036	0.001	1.000	0.979	0.006

**Table 5:** *p*-Values of Significantly Different Attributes using Choose-All-That-Apply (CATA) for Correct Response of the 2-AFC Sensory Analysis on Day 1.

Sample *p*-Value	Savory	Mouthfeel	Rancidity	Firmness	Meaty Flavor
Beef	<0.0001	<0.0001	1.000	<0.0001	<0.0001
Turkey	<0.005	<0.0001	<0.0001	<0.0001	<0.0001

**Table 6:** Mean Score from Consumer Acceptability Test for Attributes in Beef and Turkey Meatballs on Day 14.

Meat	Attribute	Control	EDTA	AHM10	AHPI10	*p*-Value
Beef	Appearance	6.26	6.05	6.12	6.04	0.766
	Aroma	5.78	6.00	5.81	5.68	0.506
	Meat Flavor	5.64	5.85	6.01	5.54	0.227
	Texture	5.96	5.60	5.94	5.70	0.376
	Overall Opinion	5.72	5.64	5.85	5.41	0.335
Turkey	Appearance	4.98	4.96	5.34	5.10	0.400
	Aroma	5.61	5.42	5.43	5.51	0.835
	Meat Flavor	5.73	5.66	5.84	5.31	0.162
	Texture	5.11	4.68	5.38	4.99	0.099
	Overall Opinion	5.46	5.14	5.63	5.19	0.177

Values ranged from 1 to 9 with 1 being “dislike extremely” and 9 being “like extremely”.

**Table 7:** Off Flavor Presence (%), Off Flavor Intensity Score * and Percentage (%) of Panelists that Would Eat the Beef or Turkey Product Again in the Consumer Acceptability Test on Day 14.

	Sample	Control	EDTA	AHM10	AHPI10	*p*-Value
Off-Flavor Presence (%)	Beef	39 ^b^	30 ^bc^	24 ^c^	49 ^a^	0.000
	Turkey	25	24	26	30	0.680
Off Flavor Intensity	Beef	7.83 ± 3.77	7.93 ± 4.22	6.69 ± 4.53	7.78 ± 4.11	0.689
	Turkey	7.26 ± 3.66	6.97 ± 3.66	8.01 ± 4.06	6.66 ± 4.10	0.647
Panelist (%) Eat Again	Beef	53	49	62	46	0.100
	Turkey	53	45	59	47	0.110

* Off-flavor intensity values ranged from 0–15 on a line scale with 0 being not intense at all and 15 being extremely intense. Values followed by different superscripts are significantly different.

## Data Availability

The original contributions presented in this study are included in the article. Further inquiries can be directed to the corresponding author.
